# Supplementary material for: A plausible mechanism for longitudinal lock-in of the plant cortical microtubule array after light-induced reorientation
Source: Quant Plant Biol. 2021 May 18;2:e9. doi: 10.1017/qpb.2021.9 (PMC10095967; doi:10.1017/qpb.2021.9)
Supplement: Supplementary file 1 [file qpbsup.zip › S2632882821000096sup001.pdf]

SUPPLEMENTARY INFORMATION

S1. STEADY-STATE SOLUTION FOR THE MICROTUBULE LENGTH DISTRIBUTION

Dynamic equations

Let $m^{\tau}(t,l)$ be the probability distribution of the length of microtubules in the state τ at time t. Following Tindemans and Mulder [Tindemans and Mulder 2010], it is possible to show that the dynamic equations that govern the model are

$$\begin{aligned}\frac{\partial}{\partial t}m_{\parallel}^{+}(t,l) =& -\alpha V^{+}(L_f)\frac{\partial}{\partial l}m_{\parallel}^{+}(t,l) - r_c m_{\parallel}^{+}(t,l) + r_r m_{\parallel}^{-}(t,l)\\ &- q\, r_s\, l\, L_{\perp}\, m_{\parallel}^{+}(t,l) + q\, r_s\left(1+p^{+}\right)L_{\perp}\left[M_{\parallel}^{+}(t,l)+M_{\parallel}^{-}(t,l)\right]\\ &+ q\, r_s L_{\perp} M_{\parallel}^{+}(t,l),\end{aligned} \tag{S1}$$

$$\begin{aligned}\frac{\partial}{\partial t}m_{\parallel}^{-}(t,l) =& \, v^{-}\frac{\partial}{\partial l}m_{\parallel}^{-}(t,l) - r_r m_{\parallel}^{-}(t,l) + r_c m_{\parallel}^{+}(t,l)\\ &- q\, r_s\, l\, L_{\perp}\, m_{\parallel}^{-}(t,l) + q\, r_s\left(1-p^{+}\right)L_{\perp}\left[M_{\parallel}^{+}(t,l)+M_{\parallel}^{-}(t,l)\right]\\ &+ q\, r_s L_{\perp} M_{\parallel}^{-}(t,l),\end{aligned} \tag{S2}$$

$$\begin{aligned}\frac{\partial}{\partial t}m_{\perp}^{+}(t,l) =& -V^{+}(L_f)\frac{\partial}{\partial l}m_{\perp}^{+}(t,l) - r_c m_{\perp}^{+}(t,l) + r_r m_{\perp}^{-}(t,l)\\ &- (1-q)\, r_s\, l\, L_{\parallel}\, m_{\perp}^{+}(t,l)\\ &+ (1-q)\, r_s\left(1+p^{+}\right)L_{\parallel}\left[M_{\perp}^{+}(t,l)+M_{\perp}^{-}(t,l)\right]\\ &+ (1-q)\, r_s L_{\parallel} M_{\perp}^{+}(t,l),\end{aligned} \tag{S3}$$

$$\begin{aligned}\frac{\partial}{\partial t}m_{\perp}^{-}(t,l) =& \, v^{-}\frac{\partial}{\partial l}m_{\perp}^{-}(t,l) - r_r m_{\perp}^{-}(t,l) + r_c m_{\perp}^{+}(t,l)\\ &- (1-q)\, r_s\, l\, L_{\parallel}\, m_{\perp}^{-}(t,l)\\ &+ (1-q)\, r_s\left(1-p^{+}\right)L_{\parallel}\left[M_{\perp}^{+}(t,l)+M_{\perp}^{-}(t,l)\right]\\ &+ (1-q)\, r_s L_{\parallel} M_{\perp}^{-}(t,l),\end{aligned} \tag{S4}$$

with boundary conditions

$$\alpha V^{+}(L_f)\, m_{\parallel}^{+}(t,0) = R_n^{\parallel}\left(L_f, L_{\parallel}\right), \tag{S5}$$

$$V^{+}(L_f)\, m_{\perp}^{+}(t,0) = R_n^{\perp}\left(L_f, L_{\perp}\right), \tag{S6}$$

$$\lim_{l\to\infty} m_{\parallel/\perp}^{-}(t,l) = 0, \tag{S7}$$

and initial conditions from the steady-state solution of the Dogterom-Leibler model, i.e.,

$$m_{\parallel}^{+}(0,l) = 0, \tag{S8}$$

$$m_{\parallel}^{-}(0,l) = 0, \tag{S9}$$

$$m_{\perp}^{+}(0,l) = \frac{R_n(L_f)}{V^{+}(L_f)}\exp\left[-\left(\frac{r_c}{V^{+}(L_f)} - \frac{r_r}{v^{-}}\right)l\right], \tag{S10}$$

$$m_{\perp}^{-}(0,l)=\frac{R_n(L_f)}{v^-}\exp\left[-\left(\frac{r_c}{V^+(L_f)}-\frac{r_r}{v^-}\right)l\right]. \tag{S11}$$

It is convenient to underline that the total amount of tubulin polarized in either of the two directions is linked to the microtubule length distributions through the equations

$$L_{\parallel}(t)=\int_0^{\infty}dl\,l\left[m_{\parallel}^{+}(t,l)+m_{\parallel}^{-}(t,l)\right], \tag{S12}$$

$$L_{\perp}(t)=\int_0^{\infty}dl\,l\left[m_{\perp}^{+}(t,l)+m_{\perp}^{-}(t,l)\right], \tag{S13}$$

i.e., the total amount of tubulin used by longitudinal/transverse microtubules is the first moment of the total length distribution of longitudinal/transverse microtubules.

Steady-state solution

The dependency of the growing speed of microtubules on the amount of free tubulin L_f in the pool implies that the microtubule length distribution eventually reaches the steady-state. Here, in order to find an analytical solution, we make the further assumption that microtubules undergo complete depolymerization suddenly after a catastrophe, i.e.,

$$\frac{\langle l\rangle}{v^-}\ll\frac{1}{r_r}, \tag{S14}$$

where $\langle l\rangle$ is the mean length of a microtubule in the system. In this limit we can identify all microtubules with the growing microtubules and, therefore, to ease the notation we remove the label + from all microtubule distributions. Thus, Eqs. (S1-S4) are replaced by

$$0=-\alpha V^+\frac{d}{dl}m_{\parallel}(l)-r_c m_{\parallel}(l)-q\,r_s\,l\,L_{\perp}\,m_{\parallel}(l)+q\,r_s\left(1+p^+\right)L_{\perp}M_{\parallel}(l), \tag{S15}$$

$$0=-V^+\frac{d}{dl}m_{\perp}(l)-r_c m_{\perp}(l)-(1-q)\,r_s\,l\,L_{\parallel}\,m_{\perp}(l)+(1-q)\,r_s\left(1+p^+\right)L_{\parallel}M_{\perp}(l). \tag{S16}$$

If we differentiate by l the last two equations we obtain

$$\alpha V^+\frac{d^2}{dl^2}m_{\parallel}(l)+\left(r_c+q\,r_sL_{\perp}\,l\right)\frac{d}{dl}m_{\parallel}(l)+q\,r_sL_{\perp}\left(2+p^+\right)m_{\parallel}(l)=0, \tag{S17}$$

$$V^+\frac{d^2}{dl^2}m_{\perp}(l)+\left(r_c+(1-q)\,r_sL_{\parallel}\,l\right)\frac{d}{dl}m_{\perp}(l)+(1-q)\,r_sL_{\parallel}\left(2+p^+\right)m_{\perp}(l)=0. \tag{S18}$$

As $L_{\parallel/\perp}=\int_0^{\infty}dl\,m_{\parallel/\perp}(l)$, Eqs. (S17) and (S18) are coupled second order differential equations, and they are also linked to Eq. (1) through $V^+(L_f)$. However, L_f, $L_{\parallel}$, and $L_{\perp}$ do not depend on l, hence Eqs. (S17) and (S18) can be in principle solved, with solutions that depend on the total amount of tubulin used by the other population and on the free tubulin. We re-write the two equations in a more elegant way as

$$\frac{d^2}{d\lambda^2}\mu_{\parallel}(\lambda)+\left(1+q\,\sigma\Lambda_{\perp}\,\lambda\right)\frac{d}{d\lambda}\mu_{\parallel}(\lambda)+q\,\sigma\Lambda_{\perp}\left(2+p^+\right)\mu_{\parallel}(\lambda)=0, \tag{S19}$$

$$\frac{d^2}{d\lambda^2}\mu_{\perp}(\lambda)+\left(1+(1-q)\,\sigma\Lambda_{\parallel}\,\lambda\right)\frac{d}{d\lambda}\mu_{\perp}(\lambda)+(1-q)\,\sigma\Lambda_{\parallel}\left(2+p^+\right)\mu_{\perp}(\lambda)=0, \tag{S20}$$

where we introduce the non-dimensional quantities

$$\lambda=\frac{r_c}{V^+}l, \tag{S21}$$

$$\Lambda_{\parallel/\perp} = \frac{r_c}{V^+} L_{\parallel/\perp}, \tag{S22}$$

$$\mu_{\parallel/\perp} = \frac{V^+}{R_n^{\parallel/\perp}} m_{\parallel/\perp}, \tag{S23}$$

$$\sigma = \frac{r_s \left(V^+\right)^2}{r_c^3}, \tag{S24}$$

and we incorporate the factor α in the parameter V^+ for longitudinal microtubules. Notice that, with this non-dimensionalization, all parameters of the model - including the independent variable λ, are functions of L_f as they depend on $V^+ (L_f)$.

To solve Eq. (S19), we first notice that the asymptotic solution for large λ decays as $\exp\left(-\frac{1}{2} q\sigma\Lambda_\perp \lambda^2 - \lambda\right)$. Therefore, we suppose there exists a function $\xi_\parallel(\lambda)$ such that

$$\mu_\parallel(\lambda) = \exp\left(-\frac{1}{2} q\sigma\Lambda_\perp \lambda^2 - \lambda\right) \xi_\parallel(\lambda). \tag{S25}$$

If we plug this in Eq. (S19) we obtain

$$\frac{d^2}{d\lambda^2}\xi_\parallel(\lambda) - \left(1 + q\,\sigma\Lambda_\perp\,\lambda\right)\frac{d}{d\lambda}\xi_\parallel(\lambda) + q\,\sigma\Lambda_\perp\left(1+p^+\right)\xi_\parallel(\lambda). \tag{S26}$$

If we change variable as $x = \left(1 + q\,\sigma\Lambda_\perp\lambda\right)/\sqrt{2q\,\sigma\Lambda_\perp}$, last equation becomes

$$\frac{d^2}{dx^2}\xi_\parallel(x) - 2x\frac{d}{dx}\xi_\parallel(x) + 2\left(1+p^+\right)\xi_\parallel(x), \tag{S27}$$

i.e., the Hermite equation [Courant and Hilbert 1989], the solution of which is the Hermite function

$$\xi_\parallel(x) = H_{1+p^+}(x). \tag{S28}$$

Therefore, the full solution for $\mu_\parallel$ becomes

$$\mu_\parallel(\lambda) = \frac{H_{1+p^+}\left(\left(1+q\,\sigma\Lambda_\perp\lambda\right)/\sqrt{2q\,\sigma\Lambda_\perp}\right)}{H_{1+p^+}\left(1/\sqrt{2q\,\sigma\Lambda_\perp}\right)} \exp\left[-\frac{1}{2}q\sigma\Lambda_\perp\lambda^2 - \lambda\right], \tag{S29}$$

and, similarly,

$$\mu_\perp(\lambda) = \frac{H_{1+p^+}\left(\left(1+(1-q)\,\sigma\Lambda_\parallel\lambda\right)/\sqrt{2\,(1-q)\,\sigma\Lambda_\parallel}\right)}{H_{1+p^+}\left(1/\sqrt{2\,(1-q)\,\sigma\Lambda_\parallel}\right)} \exp\left[-\frac{1}{2}\,(1-q)\,\sigma\Lambda_\parallel\lambda^2 - \lambda\right]. \tag{S30}$$

Eqs. (S29) and (S30) highlight a peculiar property of the model. Indeed, if $p^+ \neq 0$, both denominators of the two distribution can be identically 0. In other words, there exist two values of σ such that $H_{1+p^+}\left(1/\sqrt{2q\,\sigma\Lambda_\perp}\right) = 0$ or $H_{1+p^+}\left(1/\sqrt{2\,(1-q)\,\sigma\Lambda_\parallel}\right) = 0$, with the consequence that the number of longitudinal/transverse microtubules diverges. In the analytically tractable $p^+ = 1$ case these values are $\sigma = \frac{1}{q\Lambda_\perp}$ for the divergence of the longitudinal, and $\sigma = \frac{1}{(1-q)\Lambda_\parallel}$ for the divergence of the transverse microtubules.

S2. THE INITIAL TRANSVERSE ARRAY

To keep with the assumption coming from the experiments that initially all cortical microtubules are directed transversely to the growth direction of the cell, we build the initial array by considering dispersed and microtubule-based nucleation possible only in the transverse direction. In other words, for the creation of the initial array, we impose

$$R_n^\parallel = 0, \tag{S31}$$

$$R_n^{\perp} = r_n \frac{L_f^a}{L_f^a + L_v^a} = R_n \left(L_f \right). \tag{S32}$$

Therefore, the dynamics of the initial array is fully described by the Dogterom-Leibler model. Furthermore, the dependency of the growing speed of microtubules on the amount of free tubulin L_f in the pool implies that the microtubule length distribution eventually reaches the steady-state even in the case of initially unbounded-growth microtubules [Tindemans et al. 2014]. Thus, the solution of the model is the steady-state solution of Dogterom-Leibler model. Such a solution for the distribution of the length of microtubules is the initial condition for our model, see Supplementary Information S1.

S3. DESCRIPTION OF THE SIMULATIONS

Our simulations consist of two populations of microtubules that undergo dynamic instability. The two populations interact with each other through their overall properties. The simulations initially consist of only the transverse population undergoing dynamic instability, until a steady-state is reached, i.e., after 2 10^4 sec. We refer to that moment as $t = 0$ in Figure 3 and Figure S1. Then, we allow the nucleation of longitudinal microtubules both through microtubule-based nucleation and dispersed nucleation, as well as we allow the two populations to interact through severing events. We perform a sensitivity analysis by tuning, separately, q and p^+ from 0 to 1 for 5 10^4 sec, which is a sufficient time for the system to reach a steady-state, and we calculate $M_{\|}$, $M_{\perp}$, $L_{\|}$, $L_{\perp}$, and their derived observables ($\mathcal{P}_M$, $\mathcal{P}_L$, $\mathcal{R}_M$, $\mathcal{R}_L$). For every couple (q, p^+), we average the results over $N = 10^3$ simulations.

We use a discrete-time stochastic algorithm to simulate the switching between microtubule states, the severing of microtubules, and the nucleation of new microtubules. In this way it is possible that two events such as, e.g., catastrophe and severing, can occur to the same microtubule during the same time interval. However, our choice of time interval ($\Delta t = 0.1$ sec) is such that the occurrence of two events, although theoretically possible, is highly unlikely, with a probability of a double event in a single time interval on the order of $p \approx 10^{-10} l L_{\perp}$ for a longitudinal microtubule with length l, with parameters from Table 1.

S4. POLARIZATION AND SUPPRESSION IN CASE OF NO DIFFERENCE IN THE GROWING SPEED OF DIFFERENT POPULATIONS

Here, we show that the sole asymmetry in the system consequent to the preferential severing cannot explain the full, maintained reorientation observed in the experiments. Similarly as in Result section, we perform a sensitivity analysis by separately tuning q and p^+ from 0 to 1, and we measure number and length polarization and suppression, and the time needed by the system to achieve the reorientation, averaged over $N = 10^3$ simulations. Parameters and relative numerical values used in the simulations are listed in Table 1. However, since we are considering the case in which differently oriented microtubules have the same growing speed, in this case $\alpha = 1$.

Figure 2F, 2G, 2J shows that the system does not exhibit longitudinal polarization at the equilibrium for biologically realistic p^+, i.e., for p^+ comprises between 0 and 0.25. Similarly, Figure 2H and 2I shows that in the same range of values, although we can appreciate some degree of transverse suppression, the initial transverse array does not disappear. On the other hand, Figure 2F-2J reveals the existence of two regions in the (q, p^+) plane where the reorientation occurs and it is fast, namely, when both q and p^+ are high and, surprisingly, when they are both low. While one can easily argue that a high value of both q and p^+ is associated to a greater likelihood of increasing the size of the longitudinal population and the lifetime of their individuals and, hence, the longitudinal polarization, it is more difficult to intuitively understand the behaviour of the system for low values of those probabilities. However, lower values of q and p^+ are linked to an effective shortening of the single transverse microtubules and, therefore, to a fall in their average lifetime. As a consequence, the overall length used by the transverse array shortens, and so does the number of transverse microtubules, as their nucleation is partly correlated to the length polarized in the transverse direction. Nevertheless, although narrow areas in the heat maps of Figure 2F-2J where a full and maintained reorientation of the CA occurs do exist, a comparison with Figure 2A-2E would immediately show that, in the latter case, the same areas are wider.

As the only asymmetry between the two populations of the system is due to the probability q, Figure 2F, 2G displays, as expected, the symmetry $\mathcal{P} \to -\mathcal{P}$, as $q \to 1 - q$ for both $\mathcal{P}_M$ and $\mathcal{P}_L$.

Although from these results one can conclude that the preferential severing for the longitudinal microtubules cannot explain the maintenance of the array in the longitudinal direction, at least in the biological range of values for p^+, the dynamic behaviour of the two microtubule populations for high values of q and p^+ highlights an interesting fact. Indeed, Figure 2J shows that high values of p^+ and q are associated with fast reorientation, showing that, even though

the preferential severing for longitudinal microtubules and a high probability of stabilization-after-severing are neither necessary nor sufficient to achieve CA reorientation, they are able to accelerate this process significantly.

Notice that the amount of free tubulin in the pool does not substantially change from the initial value where only transverse microtubules are present, to the final steady-state value, see Figure S1B. Curiously, Figure S1A also shows that at the start of the reorientation process, i.e., at $t = 0$, there is a sudden little drop of the number of transverse microtubules. This may be explained by the sudden change in the nucleation rate of old microtubules, as it switches from R_n to $R_n/2$ as a consequence of the imposed isotropy of the system.

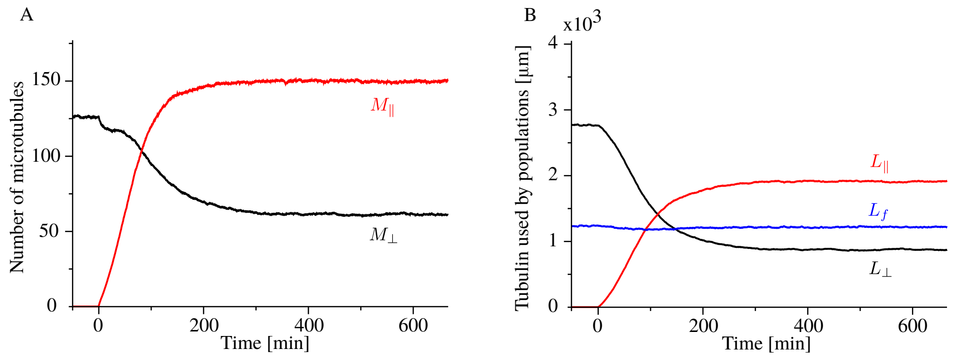

Figure S1. Time evolution of (A) longitudinal (red) and transverse (black) microtubules, and (B) tubulin used by the longitudinal population (red), the transverse population (black), and the free tubulin (blue), averaged over $N = 10^3$ simulations. Here, we used $q = 0.8$ and $p^+ = 0.6$.

S5. TIME-DEPENDENT SOLUTION OF THE MODEL WITHOUT SEVERING EVENTS

Here, we study the time scale of the reorientation of the array from the transverse to the longitudinal direction for the model discussed in the section "Analytical approach" of the main text, i.e., when no severing events can occur ($r_s = 0$) and microtubules depolymerize completely after a catastrophe ($\langle l \rangle / v^- \ll 1/r_r$). We focus on Eqs. (17) and (19), with their respective boundary conditions

$$M_{\parallel}(0) = 0 = L_{\parallel}(0) .$$

By plugging these boundary conditions in Eqs. (17) and (19) we obtain the boundary conditions for the first derivative of both $M_{\parallel}$ and $L_{\parallel}$

$$\left. \frac{dM_{\parallel}(t)}{dt} \right|_{t=0} = R_n \frac{\frac{1}{2} L_*}{L_{tot} - L_f + L_*},$$

$$\left. \frac{dL_{\parallel}(t)}{dt} \right|_{t=0} = 0.$$

Eqs. (17) and (19) can be decoupled to obtain

$$\frac{d^2 L_{\parallel}(t)}{dt^2} + 2r_c \frac{dL_{\parallel}(t)}{dt} + \left(r_c^2 - \alpha V^+ \widetilde{R}_n \right) L_{\parallel}(t) = \alpha V^+ \widetilde{R}_n \frac{1}{2} L_*, \tag{S33}$$

and

$$\frac{d^2 M_{\parallel}(t)}{dt^2} + 2r_c \frac{dM_{\parallel}(t)}{dt} + \left(r_c^2 - \alpha V^+ \widetilde{R}_n \right) M_{\parallel}(t) = r_c \widetilde{R}_n \frac{1}{2} L_*, \tag{S34}$$

where we defined

$$\widetilde{R}_n = \frac{R_n}{L_{tot} - L_f + L_*}.$$

Eqs. (S33) are second order non-homogeneous differential equations, the solutions of which are

$$
\begin{aligned}
L_{\parallel}(t) = \frac{1}{2} L_* \frac{\alpha V^+ \widetilde{R}_n}{r_c^2 - \alpha V^+ \widetilde{R}_n} &+ \frac{1}{2} L_* \frac{\sqrt{\alpha V^+ \widetilde{R}_n}}{2\left(r_c^2 - \alpha V^+ \widetilde{R}_n\right)} \\
&\times \left[\left(r_c - \sqrt{\alpha V^+ \widetilde{R}_n} \right) e^{-\left(r_c + \sqrt{\alpha V^+ \widetilde{R}_n}\right)t} - \left(r_c + \sqrt{\alpha V^+ \widetilde{R}_n} \right) e^{-\left(r_c - \sqrt{\alpha V^+ \widetilde{R}_n}\right)t} \right],
\end{aligned}
\tag{S35}
$$

$$
\begin{aligned}
M_{\parallel}(t) = \frac{1}{2} L_* \frac{r_c \widetilde{R}_n}{r_c^2 - \alpha V^+ \widetilde{R}_n} &+ \frac{1}{2} L_* \frac{\widetilde{R}_n}{2\left(r_c^2 - \alpha V^+ \widetilde{R}_n\right)} \\
&\times \left[\left(\sqrt{\alpha V^+ \widetilde{R}_n} + r_c \right) e^{-\left(r_c - \sqrt{\alpha V^+ \widetilde{R}_n}\right)t} - \left(\sqrt{\alpha V^+ \widetilde{R}_n} - r_c \right) e^{-\left(r_c + \sqrt{\alpha V^+ \widetilde{R}_n}\right)t} \right].
\end{aligned}
\tag{S36}
$$

These expressions define the time scale of the reorientation process, i.e.

$$
t_{reor} \propto \left(r_c - \sqrt{\alpha V^+ \widetilde{R}_n} \right)^{-1}. \tag{S37}
$$

To highlight all dependencies of t_{reor} on the model parameters, we can conveniently rewrite $\alpha V^+ \widetilde{R}_n$ by expressing all quantities as functions of L_f. We find

$$
\alpha V^+ \widetilde{R}_n = \alpha v^+ r_n \frac{L_f}{L_f + L_v} \frac{L_f^a}{L_f^a + L_v^a} \frac{1}{L_{tot} - L_f + L_*} = r_c^2 \frac{\alpha}{\alpha + \frac{1}{2} \frac{L_*}{L_{tot} - L_f} (\alpha + 1)}.
$$

Thus, the time scale of the reorientation can be written now as

$$
t_{reor} \propto \left[r_c \left(1 - \sqrt{\frac{\alpha (L_{tot} - L_f)}{\alpha (L_{tot} - L_f) + \frac{1}{2} L_* (\alpha + 1)}} \right) \right]^{-1}. \tag{S38}
$$

L_f is weakly dependent on r_c. As a consequence, Eq. (S38) reveals that the time scale of the reorientation is inversely proportional to the catastrophe rate. The interpretation of this counter-intuitive result, is that every time a microtubule undergoes a catastrophe, it releases to the free tubulin pool an amount of tubulin equal to its length. Therefore, the amount of building material available for the new array increases with higher rate, and so does the speed of reorientation.

S6. POLARIZATION AND TRANSVERSE SUPPRESSION IN THE MODEL WITHOUT SEVERING EVENTS

We calculate the reorientation polarization and transverse suppression for the model discussed in the section "Analytical approach" of the main text, i.e., when no severing events can occur ($r_s = 0$) and microtubules depolymerize completely after a catastrophe ($\langle l \rangle / v^- \ll 1/r_r$). From Eqs. (30-33) we obtain

$$
\mathcal{P}_M = \frac{M_{\parallel} - M_{\perp}}{M_{\parallel} + M_{\perp}} = \frac{(\alpha - 1)\left(1 - \frac{1}{2}\lambda_*\right)}{(1 + \lambda_*)\left[(\alpha - 1) + \frac{1}{2}\lambda_* (\alpha + 1)\right]}, \tag{S39}
$$

and

$$
\mathcal{P}_L = \frac{L_{\parallel} - L_{\perp}}{L_{\parallel} + L_{\perp}} = \frac{(\alpha - 1)\left(1 + \frac{1}{2}\lambda_*\right)}{(\alpha - 1) + \frac{1}{2}\lambda_* (\alpha + 1)}. \tag{S40}
$$

As expected, both reorientation parameters are close to 1 when λ_* is small, see Figure S2A, S2B, whilst they rapidly decay to 0 for higher values of λ_*.

Similarly, we can calculate the transverse suppression:

$$
\mathcal{R}_M = \frac{M_{\perp}^0 - M_{\perp}}{M_{\perp}^0 + M_{\perp}} = \frac{\left(\frac{1}{2}\lambda_*\right)^2 (\alpha + 1) + \frac{1}{2}\lambda_* (2\alpha - 1) + (\alpha - 1)}{3\left(\frac{1}{2}\lambda_*\right)^2 (\alpha + 1) + \frac{1}{2}\lambda_* (4\alpha - 1) + (\alpha - 1)}, \tag{S41}
$$

and

$$\mathcal{R}_L = \frac{L_\perp^0 - L_\perp}{L_\perp^0 + L_\perp} = \frac{\alpha\left(1+\frac{1}{2}\lambda_*\right) - 1}{\alpha\left(1+\frac{1}{2}\lambda_*\right) - 1 + \lambda_*}. \tag{S42}$$

Figure S2C, S2D shows that in the case of the transverse suppression the effect of the bias in the speed is to almost completely remove the initial transverse array.

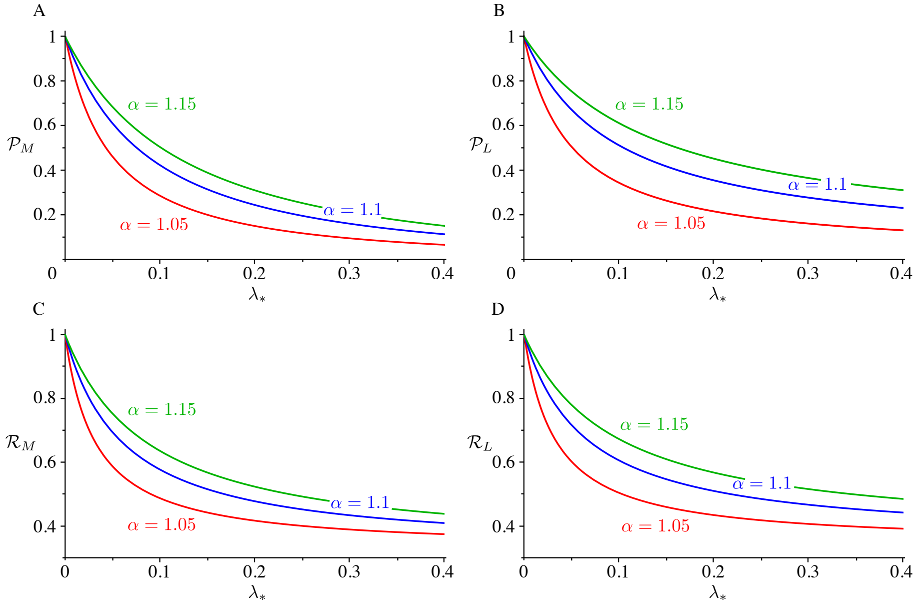

Figure S2. Polarization for (A) the microtubules and (B) the tubulin, and suppression for (C) microtubules number and (D) length as a function of the propensity length for dispersed nucleation for $\alpha = 1.05$ (red), $\alpha = 1.1$ (blue), and $\alpha = 1.15$ (green).

Courant, R. and Hilbert, D. (1989). *Methods of Mathematical Physics*. Wiley-Interscience.

Tindemans, S. H., Deinum, E. E., Lindeboom, J. J., and Mulder, B. M. (2014). Efficient event-driven simulations shed new light on microtubule organization in the plant cortical array. *Front. Phys.*, 2:1–15.

Tindemans, S. H. and Mulder, B. M. (2010). Microtubule length distributions in the presence of protein-induced severing. *Phys. Rev. E*, 81(3):031910.
